# Differences in Health Care Expenditures by Cancer Patients During Their Last Year of Life: A Registry-Based Study

**DOI:** 10.3390/curroncol31100462

**Published:** 2024-10-16

**Authors:** Peter Strang, Max Petzold, Linda Björkhem-Bergman, Torbjörn Schultz

**Affiliations:** 1 Department of Oncology-Pathology, Karolinska Institutet, Stockholms Sjukhem Foundation, Mariebergsgatan 22, SE 11219 Stockholm, Sweden; 2 Research and Development Department, Stockholm’s Sjukhem Foundation, Mariebergsgatan 22, SE 11219 Stockholm, Sweden; linda.bjorkhem-bergman@ki.se (L.B.-B.); torbjornschultz@gmail.com (T.S.); 3 School of Public Health and Community Medicine, Institute of Medicine, University of Gothenburg, 40530 Gothenburg, Sweden; max.petzold@gu.se; 4 Department of Neurobiology, Care Sciences and Society (NVS), Division of Clinical Geriatrics, Karolinska Institutet, 17177 Solna, Sweden

**Keywords:** cancer, palliative care, health expenditure, health care costs, health care utilization

## Abstract

Background. During the last year of life, persons with cancer should probably have similar care needs and costs, but studies suggest otherwise. Methods. A study of direct medical costs (excluding costs for expensive prescription drugs) was performed based on registry data in Stockholm County, which covers 2.4 million inhabitants, for all deceased persons with cancer during 2015–2021. The data were mainly analyzed with the aid of multiple regression models, including Generalized Linear Models (GLMs). Results. In a population of 20,431 deceased persons with cancer, the costs increased month by month (*p* < 0.0001). Higher costs were mainly associated with lower age (*p* < 0.0001), higher risk of frailty, as measured by the Hospital Frailty Risk Scale (*p* < 0.0001), and having a hematological malignancy. In a separate model, where those 5% with the highest costs were identified, these variables were strengthened. Sex and socio-economic groups on an area level had little or no significance. Systemic cancer treatments during the last month of life and acute hospitals as place of death had only a moderate impact on costs in adjusted models. Conclusions. Higher costs are mainly related to lower age, higher frailty risk and having a hematological malignancy, and the effects are both statistically and clinically significant despite the fact that expensive drugs were not included. On the other hand, the costs were mainly comparable in regard to sex or socio-economic factors, indicating equal care.

## 1. Introduction

It is well known that health care expenditures are relatively high at the end of life (EOL) and that the costs increase dramatically during the last months and weeks of life regardless of diagnosis [1]. Moreover, studies show that the expenses are not evenly distributed, as a limited group of so-called high-cost users account for a significant part of the total expenditure [2,3,4]. High-cost use in a general population is associated with more inpatient care and less ambulatory care [2,3].

In regard to EOL expenditures for persons with cancer, the total health care expenditures are high during the last year of life [1] and are higher for younger, as well as for married, persons [5,6], whereas some data indicate that the mean costs might be somewhat lower for women [1,6,7]. In studies on comorbidity, data are diverging, although comorbidity seems to be associated with higher costs [5,6,8].

Treatment intensity in parallel with costly treatment options provided by new targeted therapies and immunotherapies contribute to life prolongation in certain patient groups in early palliative stages [9,10,11] but may also cause severe adverse effects and high costs with few benefits in late stages [12,13]. Moreover, the potential of new drugs during the very last months of life is unclear, as most of these treatments are tested in persons with a good performance status [14], which is seldom seen in persons at the end of life [14,15]. 

For these reasons, systemic cancer treatments during the last month of life are considered as futile as they are associated with a poorer quality of life if prescribed during the last month of life with no obvious survival advantages [12,13]. The American Association American Society of Clinical Oncology (ASCO) and the European Society for Medical Oncology (ESMO) advise against such use, especially in persons with a poor performance status (Eastern Cooperative Oncology Group (ECOG) Performance status 3–4) and in situations where the patient has not responded to previous lines of treatment [14,16].

However, such treatments are still offered to some cancer patients. Such aggressive treatments are associated with higher costs whether providing traditional chemotherapy [17] or chemotherapy and targeted therapies [5], but the studies are, so far, limited in size. Regarding modern alternatives, such as immune checkpoint inhibitors, their use is controversial during the last month of life [15,16,18], as such use is associated with poorer quality of life, lower hospice enrollment and deaths in acute hospitals [15]. However, such treatments are still regularly prescribed. 

Costs for aggressive treatment regimens are not just limited to high drug costs but also to higher health care utilization both in outpatient and inpatient care. In cases of severe complications, patients may also be in need of expensive intensive care unit (ICU) treatments during the last weeks and days of life [19,20].

For types of cancer and costs, the results diverge depending on study endpoints and whether expensive drugs are included. However, regardless of study design, care costs for hematological malignancies are consistently higher than for solid cancers and, therefore, are a relevant variable in all cancer studies on expenditures [1,6,21].

Studies reveal higher care costs early in the cancer trajectory and in EOL situations, and increased health care expenditures are also related to costly drugs and to excessive acute health care utilization, e.g., emergency room (ER) visits [6,21,22]. However, two variables that are often overlooked are comorbidity and frailty, and current studies have shown a strong association between these two variables and acute health care utilization and deaths in acute hospitals [5,22,23,24,25].

In regard to cost-of-care studies, a reasonable point of departure would be that persons with advanced cancer in EOL situations probably have similar medical care needs, regardless of sex, age or socio-economic factors, and, thus, similar care costs, especially when costs for diagnosis-specific, expensive drugs are excluded. This seems not to be the case, although data are diverging. More data are needed, especially in a tax-financed health care system, which means that care costs are not dependent on individual insurances.

The aims were to study whether care expenditures increase and are evenly distributed during the last year of life in relation to age, sex, socio-economic areas of living (Mosaic), comorbidity (Charlson Comorbidity Index CCI), frailty (Hospital Frailty Risk Score, HFRS), use of systemic oncological treatments during the last month of life and acute hospitals as place of death, both as single variables and in adjusted models. We excluded the costs of expensive drugs and will present data on such expenses in a future article.

## 2. Materials and Methods

We used the Strengthening the Reporting of Observational Studies in Epidemiology (STROBE) criteria for the methods and the results [26].

### 2.1. Study Design

We based this retrospective registry data study on administrative data from the Stockholm region’s central data warehouse (the VAL data base). This data base contains descriptive data such as age, sex, socio-economic data on area level (Mosaic), International Classification of Diseases 10th edition (ICD-10) codes, nationally used KVÅ-codes (Klassifikation av Vårdåtgärder, appr. “Classification of care interventions”, performed in outpatient or inpatient care), outpatient visits, as well as periods of inpatient care.

### 2.2. Population

In an initial, broad comparison, the costs of those who died of cancer were compared with those who died of other causes in order to place the costs of cancer care in a larger perspective (i.e., all deaths during 2015–2021, regardless of diagnosis or place of care). After this first comparison, the study was limited to people in ordinary housing who died of cancer.

Inclusion criteria (the main study group): The catchment area (Region Stockholm) covers about 2.4 million inhabitants. The study period was between the years 2015 and 2021 and included all adult persons aged 18 years or more who died with advanced cancer. As the administrative data warehouse that we used does not include formal death certificates but does provide ICD-10 codes for each outpatient visit or hospital stay, the ICD-10 codes were used as follows to define advanced cancer at the EOL: (a) an ICD-10 code of cancer as the main diagnosis and a concomitant distant metastasis (secondary malignant neoplasm); (b) an ICD-10 diagnosis of a malignant brain tumor; (c) an ICD-10 diagnosis of a hematological malignancy. The criteria (b) and (c) were added as the requirement for distant metastases in criteria (a) is not applicable for these malignancies.

Exclusion criteria: Persons with advanced cancer who were nursing home residents were entered in an initial comparison, but not included in the main study group, in order to create a homogeneous group of persons with advanced cancer in ordinary accommodation. 

### 2.3. Variables

Simulated (SIM) costs, i.e., relative costs, were calculated for the following variables: age groups, sex, socio-economic Mosaic groups (socio-economics at area level), comorbidity as measured by the Charlson Comorbidity Index (CCI), risk of frailty as measured by the Hospital Frailty Risk Score (HFRS), cancer type (hematological malignancies vs. solid cancers), aggressive cancer treatment (systemic cancer treatment during the last month of life Yes/No) and acute hospitals as place of death (Yes/No). Relative costs were also calculated in multivariable models using Generalized Linear Models (GLMs) in order to control for confounding variables.

Simulated costs (SIM costs): The actual costs for Region Stockholm, based on the cost level in 2019, have been allocated to respective areas of care (somatic specialist care, e.g., hospital inpatient and outpatient care; geriatrics; psychiatry; primary care; other forms of care). The total costs (appr. SEK 60 billion or EUR 5.26 billion (EUR 1 = SEK 11.40) are included, except for costs for expensive drugs that are prescribed on an individual basis (a cost of appr. SEK 5.7 billion (or EUR 0.5 billion) for the region, as these drugs are subsidized to almost 100% by the region) and except for minor internal costs (appr. SEK 0.8 billion (EUR 0.07 billion)). 

The costs within each area of care are then distributed based on (a) weighted Diagnosis-Related Groups (DRGs) (about 50% of all costs) or (b) the type of visits and the type of inpatient care using weighted keys recommended by the National Board of Health and Welfare (Socialstyrelsen) in association with the Swedish Association of Local Authorities and Regions (Sveriges Kommuner och Regioner SKR) (about 40% of the allocated costs) [27]. In the remaining 10% of all costs (c), the actual costs that were charged according to the final accounts were booked, which allows for the calculation of the average cost per patient. For an individual patient, these average costs are possible to follow throughout different areas of care.

The Health Care Administration (Hälso- och sjukvårdsförvaltningen HSF) at Region Stockholm HSF uses the SIM-costs model for routine management and control of the economy in Region Stockholm, e.g., as a basis for budgets. This type of use of actual mean costs is recommended in regard to health expenditure studies [28].

Mosaic groups: Mosaic was initially developed as a commercial tool to understand socio-economic factors on an area level but has also been used in health care [29,30,31]. Region Stockholm (former County Council) subscribes to an applied version which allows for the division of Stockholm County into 1300 relatively homogeneous and comparable socio-economic areas. These areas are classified as Mosaic 1–3, where Mosaic 1 means that the person lives in an area with the highest socio-economic standard. The three groups are made similar in size, and the classification is based on traditional socio-economic factors such as education, income, employment and housing, as well as factors such as family situation, marital status, number of children, age, ethnicity, etc. In total, more than 40 variables are analyzed in iterative cluster analyses to define the three final groups. The rationale for the Mosaic groups is that people tend to live where other people with similar backgrounds and in similar phases of life also live.

CCI: Comorbidities are important in analyses of health care consumption, and the CCI is often used to reflect the comorbidity burden [32] in a way that enables comparisons between studies [5,6,8]. As all included patients had a main malignant diagnosis, and a majority also had metastatic disease, cancer was excluded from the calculation of the CCI. The look-back period when calculating the index was one year.

HFRS: Frailty is an emerging variable in health care studies due to frailty affecting health care consumption. However, frailty is rarely measured in larger, comprehensive cohorts. Therefore, the HFRS, which is based on 109 weighted ICD-10 diagnoses often seen in frailty, was developed as a proxy measure of frailty [33]. Thus, the HFRS does not measure actual frailty in an individual but represents the estimated risk of frailty.

Cancer type: The patients were divided into two main groups and the following comparison was made: all solid cancers versus hematological malignancies, as the latter ones are known to be associated with high costs [1,6,21]. 

Aggressive EOL cancer treatment: Receiving parenteral cancer treatments during the last month of life is considered to be aggressive care and associated with higher costs [5,34]; 

Hospital deaths: The costs for persons with emergency hospitals as the place of death are higher in some studies, possibly due to hospital deaths being associated with longer periods of inpatient care and, sometimes, with intensive care unit (ICU) care [19,20].

### 2.4. Selection Bias and Dropouts

As reporting to the data base is mandatory (primary care, as well as hospital care visits and hospitalizations) and a basis for the economic compensation of respective care units, the data are mainly complete, with only a few values being missing due to accidental mistakes (estimated to be much less than 1 percent).

### 2.5. Study Size

As all deceased with a diagnosis of advanced cancer between 2015 and 2021 were included, no power calculations were performed. 

### 2.6. Statistical Methods and Missing Data

Both medians (including interquartile range (IQR)) and means (including 95% confidence intervals (95% CI)) were calculated to give a more detailed presentation as the costs were not symmetrically distributed but had a right-skewed distribution with a limited group of high-cost users. For the same reason, nonparametric statistics were performed in the initial comparisons: Costs were compared using a Wilcoxon rank-sum test for pairwise comparisons and a Kruskal–Wallis test for multiple comparisons. Proportions were compared using chi-square tests. To investigate whether the monthly cost increase was significant from month to month, mean values with 95% CI were calculated.

In the comparison between the 5% of patients who had the highest costs versus the others, binary logistic regression models were used. 

C-statistic (equivalent to the area under the curve [AUC]) was chosen as a measure of goodness-of-fit for multiple logistic regression models. The C-statistic should be interpreted as follows: values between 0.5 and 1 are possible, and 1.0 means a perfect goodness-of-fit.

In the main analyses, Generalized Linear Models (GLMs) were used, as GLMs are recommended for analyses of data that are not normally distributed and where heteroscedasticity occurs [35]. For the analyses, we used a negative binomial distribution and a log-link function [36]. The analyses produced exponentiated coefficients of the independent variables (sex, age, etc.) with rate ratios (RRs). An RR of 1.0 means that there are no statistical associations between the independent variable and changes in cost.

The only missing data were the data regarding the Mosaic classification of 152 patients which were not substituted; the data were complete for all other variables. The statistical analyses were performed with the aid of the SAS 9.4/Enterprise guide 8.2.

### 2.7. Ethics

The Regional Ethical Review Authority (EPN 2017/1141-31) approved this study.

## 3. Results

### 3.1. Initial, Broad Comparisons Between Cancer and Non-Cancer

Initially, only persons in ordinary accommodation were included, albeit with significant differences: persons with advanced cancer had a median cost of SEK 488 thousand (mean cost SEK 580 thousand) compared with a median cost of SEK 195 thousand (mean cost SEK 312 thousand) for persons who died from non-cancer diagnoses (*p* < 0.0001), as seen in Table 1.

In a second step, costs for nursing home residents with or without a cancer diagnosis were compared. Only persons aged 65 years or more were included due to most nursing home residents, with few exceptions, being aged 65 years or older. Also, in this comparison, significant differences were found for direct medical care costs (i.e., costs for the region/county council), which reached a median cost of SEK 348 thousand and 84 thousand (mean cost of SEK 412 thousand and 169 thousand) for residents with and without a cancer diagnosis, respectively (*p* < 0.0001). However, the expenditure for municipalities for basic nursing home costs (accommodation, meals, basic health care up to the level of registered nurses) was not included in the comparison.

### 3.2. The Main Study Group: Costs for Persons in Ordinary Accomodation

In order to create a homogeneous cohort, the main study included only persons in ordinary accommodation with complete data (Mosaic data were missing for 152 persons and, thus, excluded), which amounted to 20,431 deceased patients with advanced cancer, as seen in Table 2. The mean age of the population was 71.9 years.

The median medical care cost per individual was slightly higher for women and men, SEK 498 thousand vs. SEK 479 thousand (*p* = 0.0003, Wilcoxon rank-sum test), whereas the mean costs (95% CI) (SEK 584 (576–592) thousand vs. SEK 576 (567–584) thousand) were not statistically different, Table 2. The costs were significantly related to age and had a steep gradient: while the median cost was SEK 599 thousand for persons aged 18–69 years, it was SEK 491 thousand and SEK 373 thousand for persons aged 70–79 years and 80 years or more. The costs for persons residing in the Mosaic 1 socio-economic areas (the most affluent area) were not significantly different from the expenditures of those living in the Mosaic 2 or 3 areas (*p* = 0.06), nor were the costs associated with the modified Charlson Comorbidity Index (CCI); however, highly significant differences related to the Hospital Frailty Risk Score (HFRS) were found, as the median costs for persons with low, intermediate or high risk of frailty were SEK 446–548–626, respectively (*p* < 0.0001), as seen in Table 2.

The median costs were significantly higher for persons with a hematological malignancy compared to those with solid cancers, at SEK 614 thousand vs. SEK 482 thousand (*p* < 0.0001). In regard to patients who did or did not receive systemic cancer treatment during the last month of life, the figures were SEK 546 thousand and SEK 482 thousand (*p* < 0.0001). For persons who had acute hospitals as their place of death, the median costs for the last year of life were lower than for the others, at SEK 449 thousand vs. SEK 497 thousand (*p* < 0.0001, Wilcoxon rank-sum test), whereas there was no significant difference between the mean costs (SEK 589 thousand vs. SEK 579 thousand, respectively). 

### 3.3. Monthly Progression of Costs 

**Figure 1:**
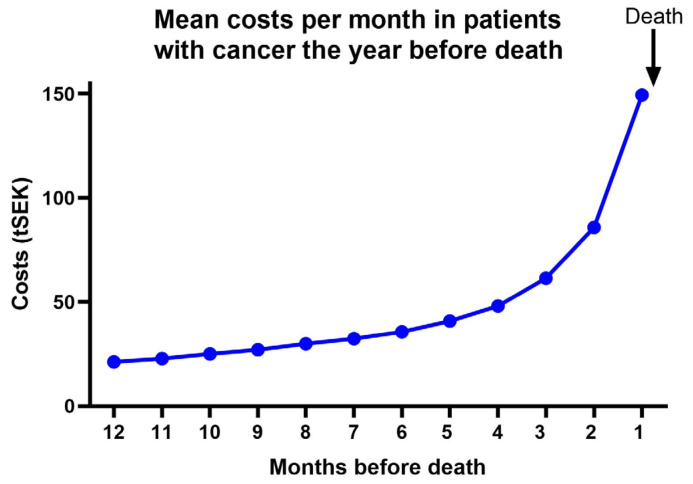
The simulated mean monthly cost per individual increased from month to month, with the most pronounced increase occurring during the last three months (EUR 1 is approximately SEK 11.40). Every monthly increase was significant (means and 95% CI).

In a separate analysis, the median (IQR) and mean (95% CI) monthly cost per individual were calculated. Both the median and the mean costs increased month by month, with a statistical difference occurring over the year (*p* < 0.0001, Kruskall–Wallis test). Every increase in the mean monthly cost was significant (i.e., the upper limit of the 95% CI did not overlap with the lower 95% CI limit of the next month), with the highest increases being seen for the last three months, Figure 1.

### 3.4. Multivariable Regression Models (Generalized Linear Models) for Costs

In order to control costs for confounding variables, an initial univariable model comparing the costs of women and men (Model A) was followed by different multivariable models, as seen in Table 3. In the first model, there were no differences in medical costs between women and men, whereas minor differences were found in the adjusted models, with a 2% higher cost. Age was an important factor, with significantly higher costs being associated with lower age in all the models, featuring RRs between 1.64 and 1.76 for the youngest age group (18–69 years), compared with the reference group (80 years or older).

Likewise, the costs for patients with intermediate or high risk of frailty according to the HFRS were significantly higher than for the reference group. The costs for hematological malignancies, compared to solid cancers, were significantly higher, with RRs of 1.49–1.51 in the different, adjusted models, whereas the costs for persons who received systemic cancer treatment during their last month of life had only marginally higher costs in the final model F (RR 1.03 (1.00–1.06), *p* < 0.05). Finally, those who died in acute hospital settings had lower costs than the others (RR 0.90 (0.88–0.82), *p* < 0.001).

### 3.5. Top 5% High-Cost Users

As the costs were not normally distributed, with some high-cost users, we also defined the characteristics of the top 5% with the highest costs with the aid of univariable and multivariable logistic regression models, as seen in Table 4. The analyses revealed that the 5% high-cost users were likely to be younger and to exhibit a high risk of frailty and/or to have a hematologic malignancy. These explanatory variables were strong in the univariable analyses and were strengthened in the adjusted, multivariable model. Undergoing a systemic cancer treatment during the last month of life was a significant variable in the univariable analysis (OR 1.31 (1.08–1.57), *p* = 0.006) but lost most of its statistical significance when controlled for other variables. Neither sex nor socio-economic Mosaic groups were significant variables.

## 4. Discussion

Our retrospective registry study on simulated (SIM) costs revealed that the health care costs for the last year of life (excluding expensive prescription drugs) were higher for persons with advanced cancer compared with those with non-cancer diagnoses. When the study was limited to persons with advanced cancer in ordinary accommodation (nursing home residents excluded), the costs were significantly higher for younger persons, those with high risk of frailty as measured by the HFRS, as well as for those with hematologic malignancies, and the costs increased month by month. In a separate analysis of the 5% top health care users, the same variables, namely low age, risk of frailty and/or having a hematologic malignancy, were even strengthened. Variables such as sex, socio-economic factors, the Charlson Comorbidity Index, systemic cancer treatments during the last month of life and hospital deaths were only weakly associated with differences in cost expenditure.

Increasing costs during the last year of life, and especially during the very last months of life, have been described previously [1] and are well in agreement with our own findings. We were able to show that the mean costs increased month by month, with especially high costs occurring during the last three months. 

In most health economic studies regarding cancer, sex has been identified as a variable related to differences in cost expenditure, with men having higher costs [1,6,7], although some studies disagree [5]. In our data, we did not see this trend; in fact, women had marginally higher cost expenditures than men in adjusted models. We do not know the reason for this, but a reasonable guess is that a tax-financed health care system promotes equal utilization of care. This assumption may also explain why we did not find any clinically significant differences in relation to socio-economic Mosaic areas the costs for persons residing in affluent areas were similar to those living in less affluent neighborhoods.

Age was highly related to costs, with a steep gradient being seen from the youngest with the highest to the oldest with the lowest costs, in agreement with other studies [5,37,38]. This finding was even more obvious when identifying the 5% high-cost users, where patients aged 18–69 years had an adjusted odds ratio (aOR) of 13.26, compared to those aged 80 years or more (reference group). Some differences may be medically justified due to treatment guidelines for cancer regularly have age restrictions, as treatments that may be beneficial for younger patients may be associated with severe side effects in the elderly. For this reason, it is expected that younger patients with cancer consume more health care during the last year of life, including clinic visits and session of inpatient care related to the treatments. Moreover, as being married is associated with higher medical cost expenditure [6], possibly due to spouses encouraging their partners to seek more care, this variable may cofound with age, as it is more likely that younger persons are married and older ones are, to a higher extent, widows or widowers. 

Comorbidities as measured by the CCI have been associated with higher costs in cancer studies [5]. This was not the case in our data, although we used the CCI in the same way as Morishima et al., i.e., we excluded cancer from the index as we only studied cancer patients in our main analysis. Instead, we used the HFRS both in the univariable and adjusted models. Comorbidities and frailty covary but are still separate constructs, as one can have comorbidities without being classified as frail [33], and vice versa. However, the HFRS is not a clinical frailty tool but rather a way of identifying persons at risk of frailty. Just like the CCI, it is based on ICD-10 diagnoses but includes no less than 109 weighted diagnoses [33]. For this reason, it bases the risk of frailty on the occurrence of multiple comorbidities.

The frailty risk as measured by the HFRS was strongly associated with higher costs, which is in line with a previous study where we have shown a significant relationship between the HFRS and unplanned emergency room visits, as well as with acute hospitals as places of death [23].

During the last year of life, the health care costs were higher for persons with hematological malignancies compared to those with solid cancer, which is in good agreement with other studies [1,6,21]. This is not surprising from a clinical point of view, as treatments of leukemias especially are very intensive and hospitalization and, in certain cases, costly ICU care are often prerequisites for such treatments. 

Patients who received systemic cancer treatments during their last month of life had higher costs than the others, but the differences were of a modest size, especially in adjusted models. This was a bit surprising as aggressive treatments near the end of life have been associated with higher costs [5]. Many authors advise against such use, as the possible benefits are very limited and the risks and costs are high [14,15,16]. We do not know the exact reasons for the modest effect size in our data and the larger effect sizes in some of the other studies, but one possible explanation is that treatment traditions may differ between countries. While we try to avoid ICU care in dying cancer patients in Swedish health care settings, the utilization of ICU care seems to be much higher in countries such as the US [19,20]. 

Surprisingly, hospital deaths were associated with somewhat lower total median costs than deaths outside acute hospitals, although the mean costs were numerically higher because the high-cost users increased the mean value. Again, we do not know the exact reason for this; however, judging from actual knowledge of the utilization of tax-financed specialized palliative care (SPC), which is offered to 79% of cancer patients during the last months of life [23], and judging from our clinical experience, those admitted to SPC have higher and more complex care needs, which may contribute to the costs, whereas those who manage to live in their own homes without the aid of SPC, and who suddenly may be in need of emergency hospitalization, may have a shorter period of medical care needs, resulting in somewhat lower costs. However, this is just a reflection, and further studies are needed to clarify this question.

Finally, we were interested in the identification of so-called high-cost users, i.e., those with the 5% highest costs, as they account for a considerable part of the total expenditure [2,3,4]. We found that the same variables that are generally associated with higher costs were the most significant ones in the group of high-cost users, namely being younger, having a higher frailty risk and being diagnosed with a hematological malignancy. Considering that expensive prescription drugs were not included in the calculations, the significant differences are a relevant point of departure for more detailed studies. 

### Strengths and Limitations

This study is based on health administrative data with large sample sizes and very few missing data. This type of study is encouraged as it has the potential to drive evidence-based palliative care practice and policy [39]. The cost model is the one that Region Stockholm (previously called Stockholm County Council) uses in real-world prognoses to forecast the development of costs, which is a strength. 

All direct costs are calculated, except for prescription drugs, which is a limitation as the new targeted therapies and immunotherapies are expensive and the costs are reported to be increasing. Thus, we cannot comment on the extent to which expensive drug costs are equitably distributed, which will be the focus of a future study. The fact that these costs were omitted means that our data are not directly comparable with other studies where these costs are included. Our main reasons for excluding the costs of expensive therapies were as follows: In the current study, we wanted to investigate whether health care costs are distributed in an equal way, and, with that objective, it is a certain advantage to focus on regular costs for outpatient and inpatient care. The use of expensive prescription drugs is guided by clinical guidelines and may, for example, depend on the presence of a gene mutation that makes a treatment possible and is, in fact, the rationale for targeted therapies. Therefore, differences in costs between different diagnoses, or between women and men, are not necessarily related to inequality but to clinical judgment.

Besides the direct medical costs, there are also huge indirect costs, mainly in the form of unpaid contributions of informal family caregivers. These costs were not the focus of this study.

Finally, the administrative data base does not include important variables such as marital status or rural/urban residence, which is a limitation.

## 5. Conclusions

The health care expenditures are higher for persons with cancer compared to those with non-cancer diagnoses. Higher medical costs are mainly associated with lower age, higher risk of frailty and having a hematological malignancy, and these associations are strengthened when studying those 5% with the highest care costs. Only minor differences were related to sex and socio-economic factors on an area level (Mosaic groups), indicating that tax-financed health care is a good basis for equal care.

## Figures and Tables

**Table 1:** General comparisons: median and mean costs per individual by SEK 1000 for persons in ordinary accommodation and for nursing home residents, stratified for non-cancer vs. cancer diagnoses (EUR 1 is approximately SEK 11.40). Persons with advanced cancer had significantly higher median costs in both comparisons.

Care Setting	Individuals, N	Median Cost In SEK 1000	Mean Cost, In SEK 1000	*p*-Value ^2^
Ordinary accommodation				<0.0001
Advanced cancer ^1^	20,431	488	580	
Non-cancer diagnoses	45,509	195	312	
Nursing home residents				<0.0001
Advanced cancer ^1^	2,625	348	412	
Non-cancer diagnoses	39,822	84	169	

^1^ Advanced cancer was defined as cancer with distant metastases, a malignant brain tumor or a malignant hematologic diagnosis. ^2^ Difference (*p*-value) in median cost per patient between those with non-cancer diagnoses and a diagnosis of advanced cancer using a Wilcoxon rank-sum test.

**Table 2:** Descriptive and clinical data for 20,431 persons with advanced cancer in ordinary accommodation. The median and mean health care expenditure costs included in the table were calculated in Swedish crowns (SEK) based on the 2019 cost level (EUR 1 is approximately SEK 11.40). The costs include all medical care costs, i.e., primary care, acute hospital care, care at geriatric and psychiatric departments.

Variable	Number (%)	Median Cost (IQR) per Individual In SEK 1000	Mean Cost (95% CI) per Individual In SEK 1000	*p*-Value
Sex, distribution Women Men	10,021 (49) 10,410 (51)	498 (312–749) 479 (302–725)	584 (576–592) 576 (567–584)	0.0003 ^2^
Age groups, distribution 18–69 years 70–79 years 80 years or more	7387 (36) 7517 (37) 5527 (27)	599 (384–882) 491 (319–723) 373 (237–554)	706 (694–719) ^4^ 564 (556–573) ^4^ 431 (423–438) ^4^	<0.0001 ^1^
Mosaic groups Group 1 Group 2 Group 3	5567 (27) 8292 (41) 6572 (32)	499 (314–747) 484 (306–729) 486 (302–736)	594 (581–606) 574 (565–583) 575 (565–585)	0.06 (ns) ^1^
CCI (cancer excluded) ^3^ 0 1 or more	9368 (46) 11,063 (54)	493 (306–732) 485 (306–736)	576 (568–584) 576 (574–591)	0.85 (ns) ^2^
HFRS Low risk (<5) Intermediate risk (5–15) High risk (>15)	12,276 7115 1040	446 (277–674) 548 (358–832) 626 (409–924)	517 (510–523) ^4^ 664 (651–676) ^4^ 749 (716–781) ^4^	<0.0001 ^1^
Type of malignancy Hematologic malignancy Other cancer forms	1233 (6) 19,198 (94)	614 (385–1056) 482 (303–724)	859 (812–906) ^4^ 562 (556–567) ^4^	<0.0001 ^2^
Systemic cancer treatment, last month of life Yes No	2093 (10) 18,338 (90)	546 (380–785) 482 (298–730)	653 (632–675) ^4^ 571 (565–577) ^4^	<0.0001 ^2^
Place of death: hospital Yes No	3696 (18) 16,735 (82)	449 (259–721) 497 (319–736)	589 (569–608) 578 (572–583)	<0.0001 ^2^

^1^ Kruskal–Wallis test. ^2^ Wilcoxon rank-sum test. ^3^ Cancer diagnoses were excluded from the calculation of the CCI, as all patients had cancer. ^4^ The 95% CI intervals did not overlap (indicating significant differences at the 0.05 level).

**Table 3:** Generalized linear models (GLMs). Model A: Sex. Model B: Sex and age. Model C: Sex, age and frailty (HFRS). Model D: Sex, age, frailty and type of malignancy (hematologic malignancy vs. other cancer forms). Model E: Sex, age, frailty (HFRS), type of malignancy and systemic cancer treatments during the last month of life. Model F: Same as model E, but with hospital added as place of death.

Variable	Model A RR (95% CI)	Model B RR (95% CI)	Model C RR (95% CI)	Model D RR (95% CI)	Model E RR (95% CI)	Model F RR (95% CI)
Women (ref. men)	1.01 (0.99–1.03)	1.00 (0.98–1.02)	1.02 * (1.00–1.02)	1.02 ** (1.01–1.04)	1.02 ** (1.01–1.04)	1.02 * (1.00–1.04)
Age (ref. ≥ 80 years)						
18–69 years		1.64 *** (1.61–1.68)	1.74 *** (1.70–1.78)	1.74 *** (1.71–1.79)	1.75 *** (1.71–1.78)	1.76 *** (1.72–1.79)
70–79 years		1.31 *** (1.28–1.34)	1.36 *** (1.33–1.39)	1.37 *** (1.35–1.40)	1.37 *** (1.34–1.40)	1.38 *** (1.35–1.41)
HFRS (ref. HFRS < 5)						
Intermediate risk (5–15)			1.33 *** (1.31–1.36)	1.31 *** (1.29–1.34)	1.31 *** (1.29–1.34)	1.32 *** (1.30–1.35)
High risk (>15)			1.66 *** (1.59–1.72)	1.63 *** (1.57–1.69)	1.63 *** (1.57–1.70)	1.65 *** (1.59–1.72)
Hematolology (ref = no)				1.49 *** (1.44–1.54)	1.48 *** (1.43–1.53)	1.51 *** (1.46–1.57)
Systemic tx (ref: no tx) ^#^					1.01 (0.99–1.04)	1.03 * (1.00–1.06)
Hospital death (ref.: not)						0.90 *** (0.88–0.92)

* = *p*-value < 0.05, ** = *p*-value < 0.01, *** = *p*-value < 0.001. ^#^ Tx = therapy.

**Table 4:** Variables associated with high-cost users, defined as the top 5% users (n = 1023 of 20,431 patients).

Variable	Univariable Analysis		Multivariable Analysis ^1^	
	OR (95% CI)	*p*-Value	aOR (95% CI)	*p*-Value
Sex				
Women	1.04 (0.92–1.18)	0.55	1.08 (0.95–1.24)	0.22
Men	Ref.		Ref.	
Age (ref. ≥ 80 years)				
18–69 years	7.80 (6.06–10.03)	<0.0001	13.26 (10.18–17.27)	<0.0001
70–79 years	3.35 (2.57–4.37)	<0.0001	4.60 (3.50–6.04)	<0.0001
≥80 years	Ref.		Ref.	
Mosaic groups				
Groups 1 + 2	1.02 (0.89–1.16)	0.83	1.06 (0.92–1.22)	0.40
Group 3	Ref.		Ref.	
HFRS (ref. HFRS < 5)				
High risk (>15)	3.97 (3.19–4.93)	<0.0001	6.83 (5.39–8.65)	<0.0001
Intermediate risk (5–15)	2.50 (2.18–2.86)	<0.0001	2.82 (2.45–3.25)	<0.0001
Low risk	Ref.		Ref.	
Malignancy (type)				
Hematological	4.45 (3.77–5.25)	<0.0001	5.38 (4.48–6.46)	<0.0001
Solid cancer	Ref.		Ref.	
Systemic treatment last month of life				
Yes	1.31 (1.08–1.57)	0.006	0.81 (0.66–0.99)	0.04
No	Ref.		Ref.	

^1^ C-statistic for the final multiple regression model was 0.74.

## Data Availability

The datasets generated and analyzed in this study are available upon reasonable request.
